# Supplementary material for: Walking modulates visual detection performance according to stride cycle phase
Source: Nat Commun. 2024 Mar 7;15:2027. doi: 10.1038/s41467-024-45780-4 (PMC10920920; doi:10.1038/s41467-024-45780-4)
Supplement: Supplementary file 1 — Supplementary Information [file 41467_2024_45780_MOESM1_ESM.docx]

# **Supplementary Information**

## ***
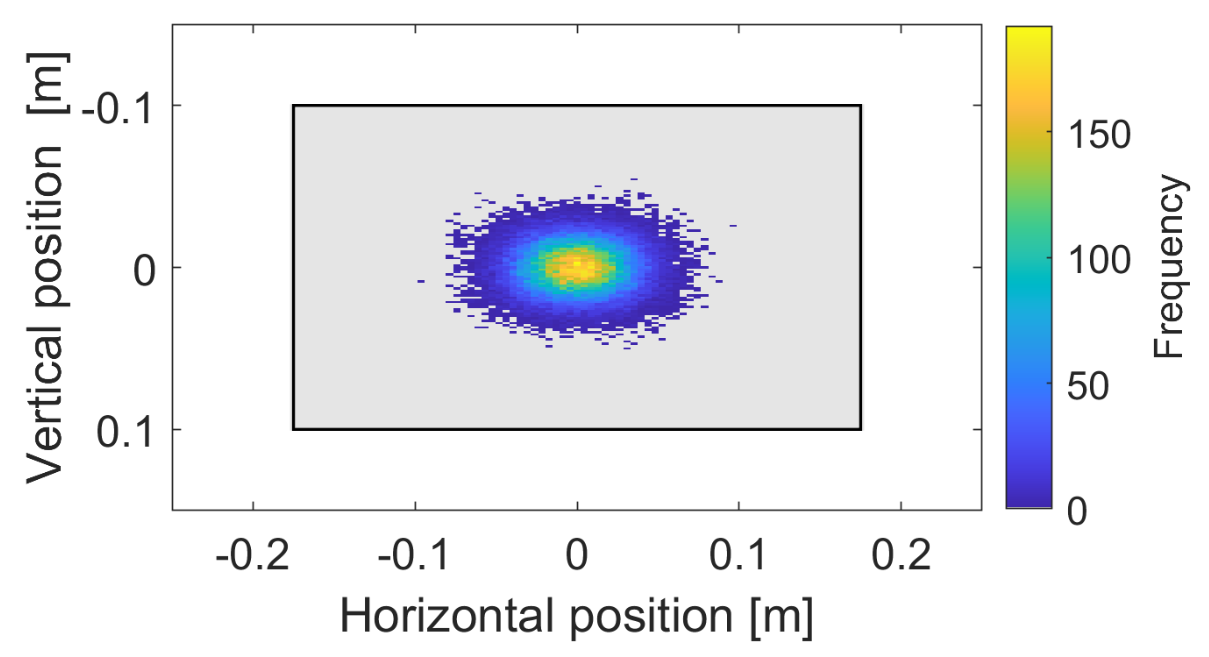
***

## **Supplementary Figure 1.** Distribution of all target locations throughout the experiment, relative to the centre of the virtual screen. Grey shaded region displays the approximate boundary of the virtual screen.


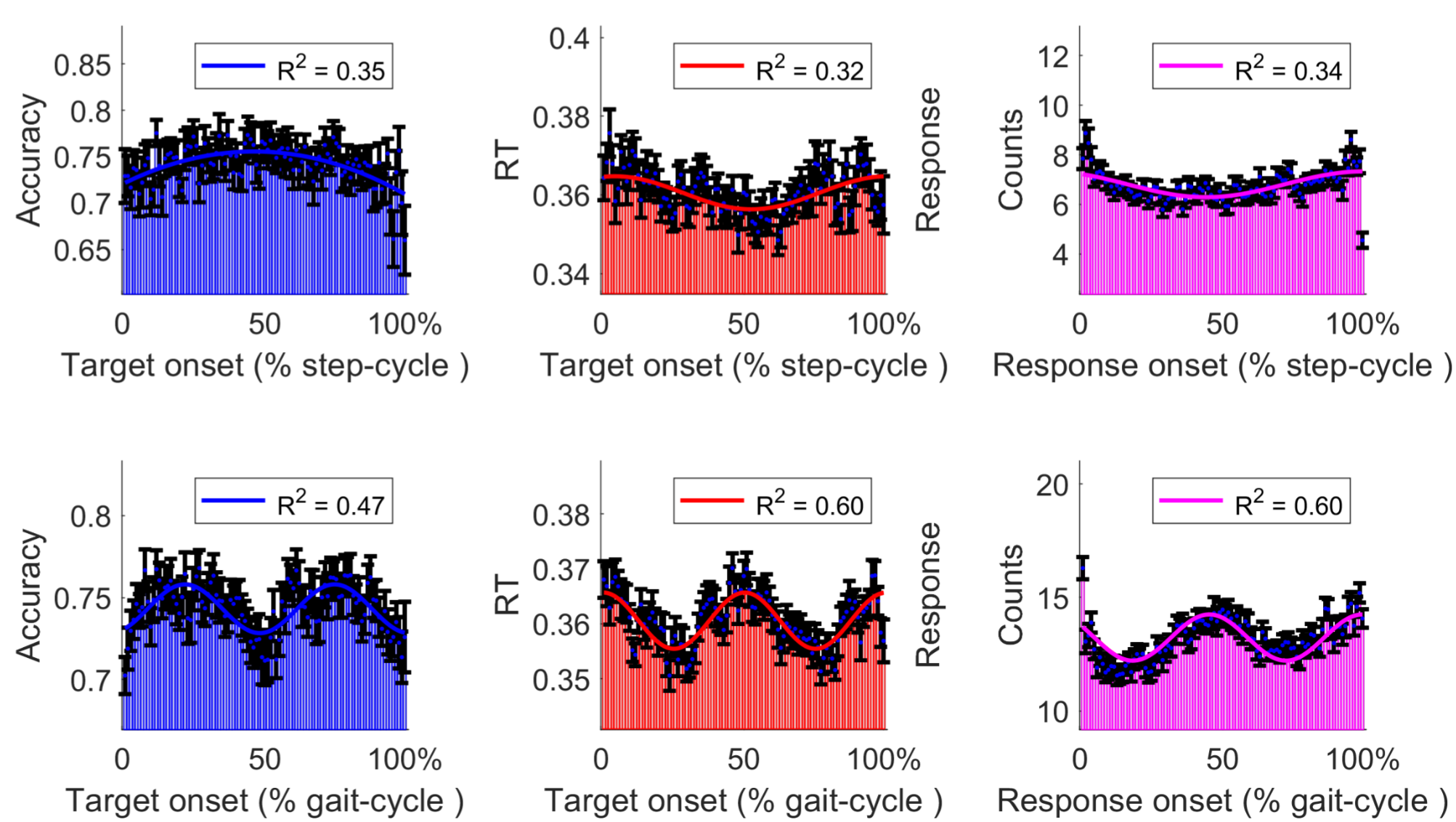


## **Supplementary Figure 2.** Oscillations in performance do not result from averaging within the linearly spaced bins in our stride cycle analyses (N=36). Both rows reproduce the main results from Figure 3, omitting this procedure, with 100 data-points in a single-step analysis (top row), and 200 data points in the stride cycle analysis (bottom row).

## ***
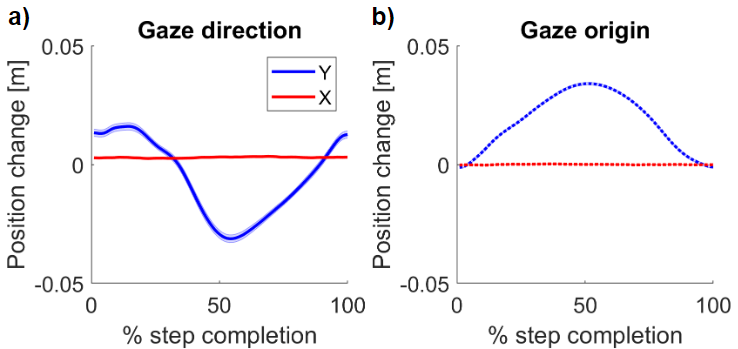
***

## **Supplementary Figure 3.** Gaze origin and gaze direction over the step-cycle (N=36). **a)** Average detrended gaze direction over a single step cycle. A clear vertical translation occurs to compensate for changes in vertical head position. **b)** Average detrended change in gaze origin over the step cycle. Like vertical head position, gaze-origin follows a smooth trajectory during the step-cycle, with no statistically significant increase in variance around the time of footfall.


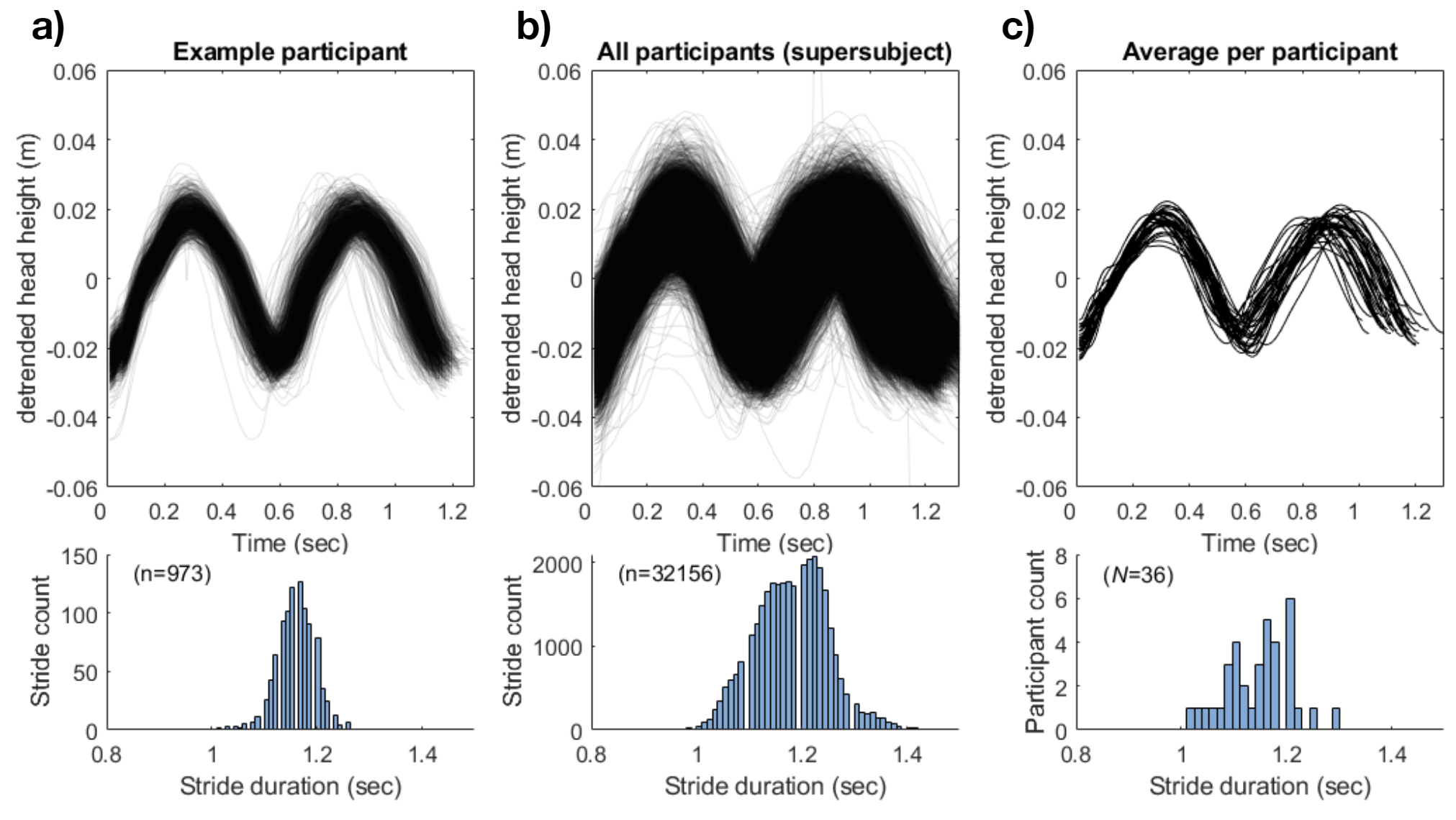


## **Supplementary Figure 4.** Example stride-cycle variability. **a)** The top panel displays the change in head-height (detrended) for all strides of a representative participant. The histogram of all stride durations (n=973) is displayed in the bottom panel. **b)** Top panel overlays all recorded strides, all participants, with the bottom panel showing the histogram of all stride durations. **c)** Top panel shows the average stride-cycle per participant (N=36), prior to resampling along the x-axis to 1-100% stride completion. The distribution of average participant stride durations is displayed in the bottom panel.

##


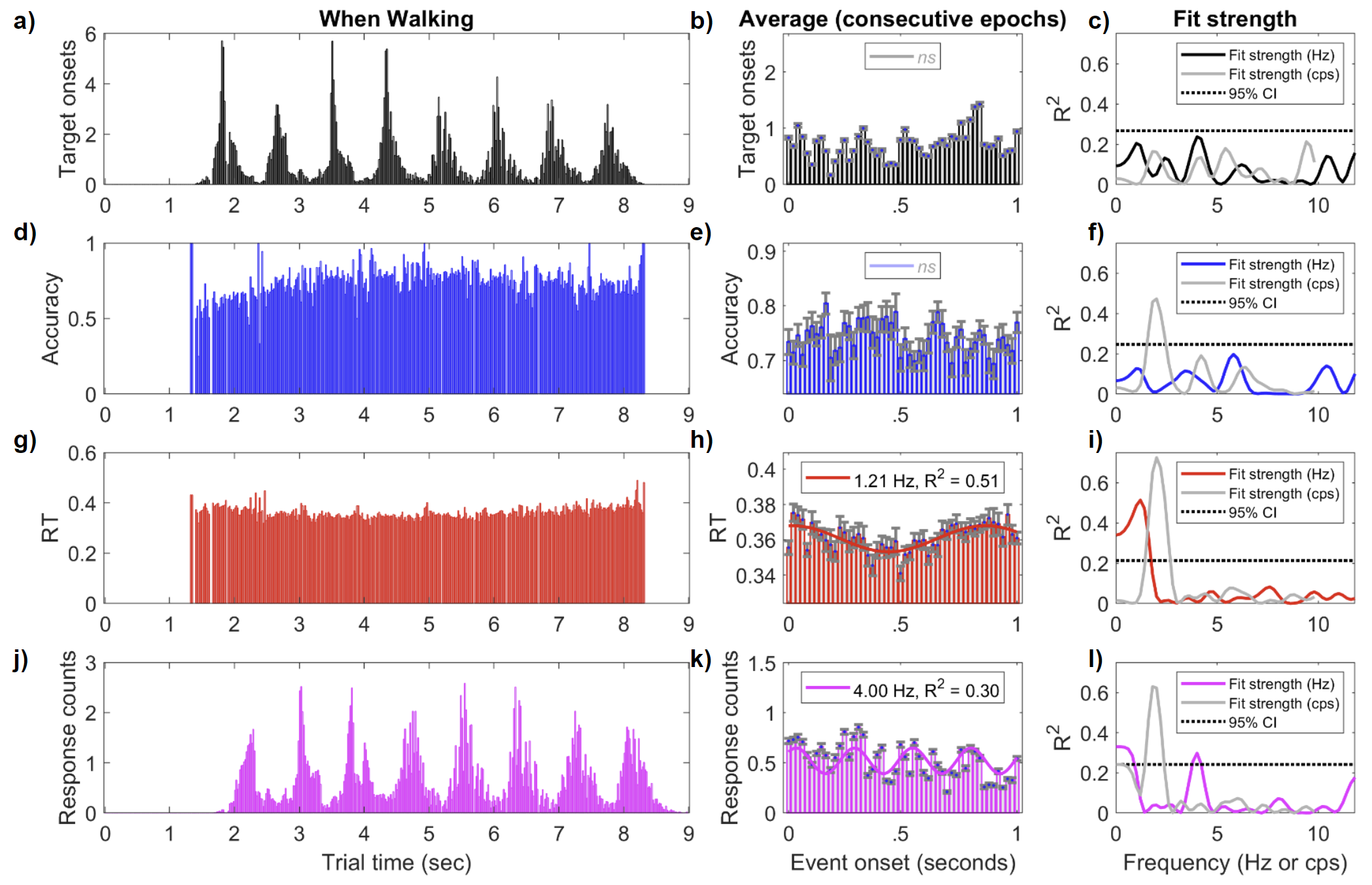


## **Supplementary Figure 5.** An analysis of perceptual oscillations when walking, aligned to clock-time (N=36). **a)** Plots the distribution of all target onsets within each 9 second trial, averaged across participants. The oscillatory structure in target onsets is expected based on our jittered target spacing. Critically, we note that despite this structure, the presentation of target onsets within a stride was approximately uniform (cf. Manuscript Figure 3C), owing to the variability of stride times across participants. **b)** Displays the average over all consecutive 1 second epochs (20 ms bins, no overlap). No statistically significant oscillations were present above the 95% CI of shuffled data. **c)** Displays the goodness-of-fit for all frequencies in Hz in black. Overlaid in grey is the equivalent strength of Fourier fits using the cycles per stride data (i.e. Manuscript Figure 3). Broken lines display the 95% CI for data aligned to clock-time. **d)** Displays the change in target accuracy over trial-time, while walking. **e)** Displays the change in accuracy on average over 1 second epochs. **f)** Demonstrates that the strength of accuracy oscillations per stride-cycle (grey) far exceeds the strength of any oscillation per second (blue). **g-i)** The same data for reaction times in red. Again, note that the fit strength when aligning to cycles-per-stride far exceeds oscillations in Hz. **j-l)** Displays the response counts per trial time, per second, and fit strength, respectively. Although there is temporal structure owing to our target spacing, fit strength during cycles per stride far exceeds fit-strength in units of time, demonstrating that perceptual oscillations are locked to the phase of the stride-cycle.


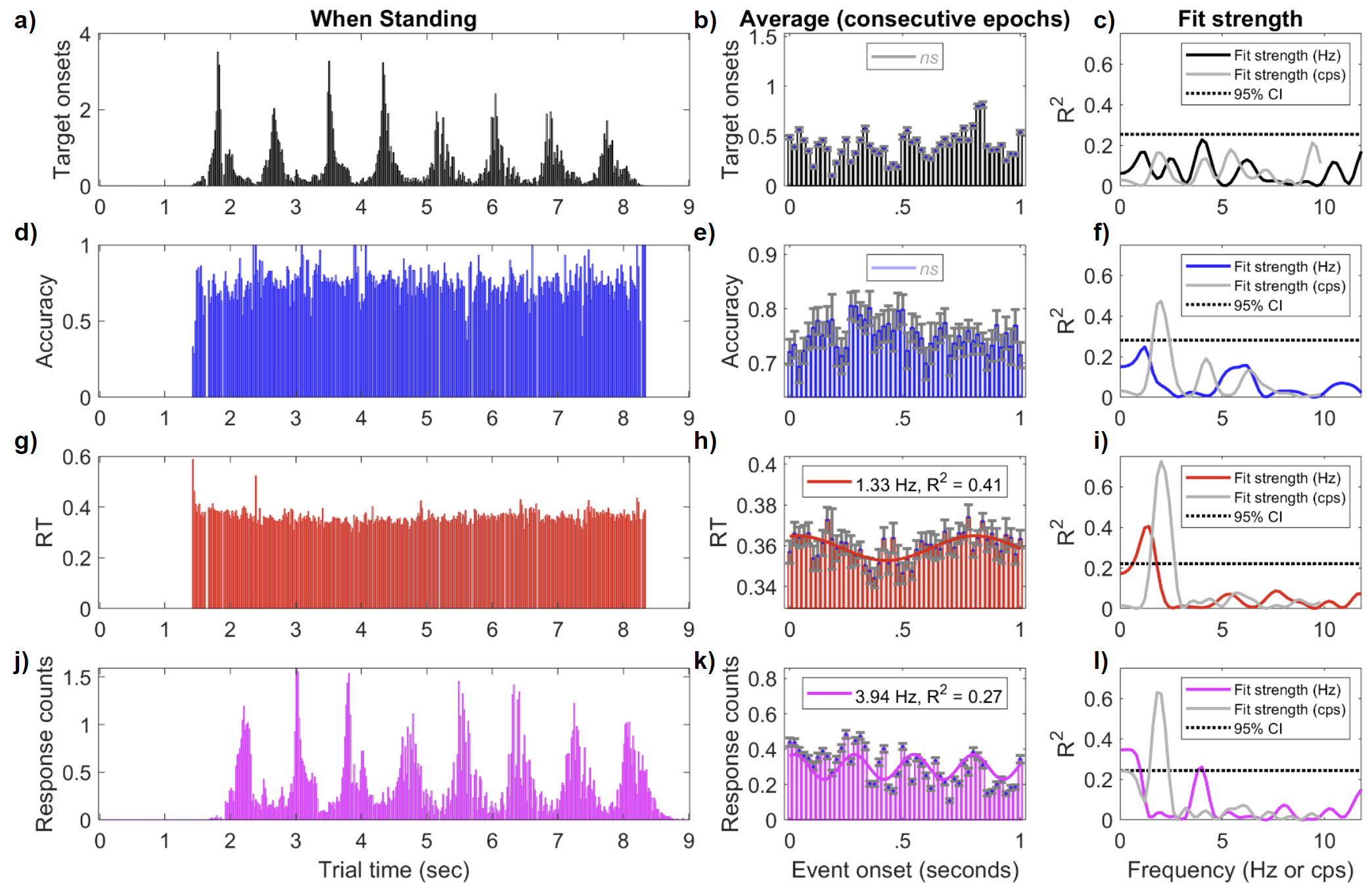


## **Supplementary Figure 6.** An analysis of perceptual oscillations when stationary, aligned to clock-time (N=36). **a)** Plots the distribution of all target onsets within each 9 second trial, averaged across participants. The oscillatory structure in target onsets is expected based on our jittered target spacing. Critically, we note that despite this structure, the presentation of target onsets within a stride was approximately uniform (cf. Manuscript Figure 3C), owing to the variability of stride times across participants. **b)** Displays the average over all consecutive 1 second epochs (20 ms bins, no overlap). No statistically significant oscillations were present above the 95% CI of shuffled data. **c)** displays the goodness-of-fit for all frequencies in Hz in black. Overlaid in grey is the equivalent strength of Fourier fits using the cycles per stride data (i.e. Manuscript Figure 3). Broken lines display the 95% CI for data aligned to clock-time. **d)** Displays the change in target accuracy over trial-time, while standing. **e)** Displays the change in accuracy on average over 1 second epochs. **f)** Demonstrates that the strength of accuracy oscillations per stride-cycle (grey) far exceeds the strength of any oscillation per second (blue). **g-i)** The same data for reaction times in red. Again, note that the fit strength when aligning to cycles per stride far exceeds oscillations in Hz. **j-l)** Displays the response counts per trial time, per second, and fit strength, respectively. Although there is temporal structure owing to our target spacing, fit strength during cycles per stride far exceeds fit-strength in units of time, demonstrating that perceptual oscillations are locked to the phase of the stride-cycle.


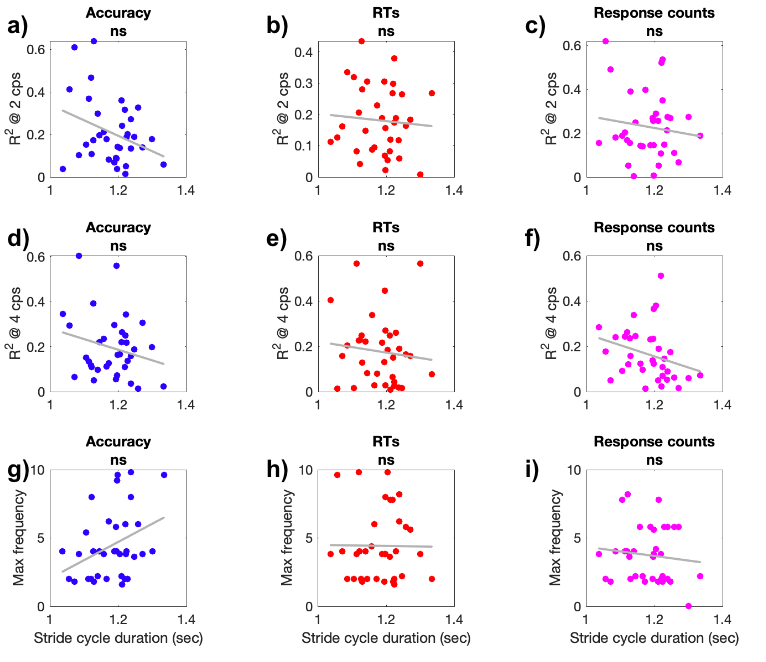


## **Supplementary Figure 7.** No statistically significant correlations between participant stride-cycle duration and the idiosyncratic oscillations in perceptual performance. **a-c)** Display scatter plots (N=36) of the strength of Fourier fits at 2 cps (max between 1.8 and 2.2 cps) and average participant stride-cycle duration, for accuracy, reaction time, and response count data respectively. **d-f)** Display the relationship between the strength of Fourier fits at 4 cps (max between 3.8 and 4.2 cps) and stride-cycle duration. **g-i)** Displays the correlation between the frequency (in cps) at which the maximum fit was reached and stride-cycle duration. All correlations are non-significant after correction for multiple comparisons.


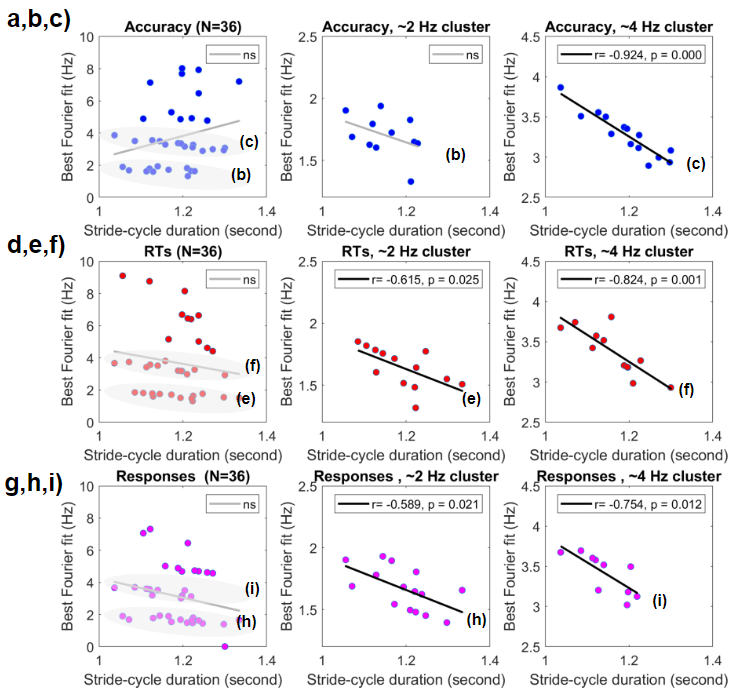


### **Supplementary Figure 8. Relationship between perceptual oscillations in Hz and stride-cycle durations.** In all panels the y-axis displays the frequency (in Hz) of the best fitting Fourier model to performance oscillations, when fitting based on stride-cycle duration (in seconds). The x-axis shows the stride-cycle duration in seconds. **a)** Displays the scatter plot and non-significant correlation (N=36) for accuracy data. Note however the clear grouping of data for oscillations at approximately 2 and 4 Hz (shaded grey regions). This grouping suggests the group-level analysis may not capture trends within each cluster (aka Simpson’s paradox). Focusing instead on the correlations within these clusters **b)** demonstrates a non-significant negative correlation between stride-duration and oscillations at approximately 2 Hz, and **c)** a significant negative correlation at approximately 4 Hz, as predicted. **d-f)** Show the same analyses on the data for oscillations in reaction times, and **g-i)** for response likelihood.
