# Supplementary material for: Walking modulates visual detection performance according to stride cycle phase
Source: Nat Commun. 2024 Mar 7;15:2027. doi: 10.1038/s41467-024-45780-4 (PMC10920920; doi:10.1038/s41467-024-45780-4)
Supplement: Supplementary file 3 — Reporting Summary [file 41467_2024_45780_MOESM3_ESM.pdf]

nature portfolio

Corresponding author(s): Matthew Davidson

Last updated by author(s): 20/01/24

Reporting Summary

Nature Portfolio wishes to improve the reproducibility of the work that we publish. This form provides structure for consistency and transparency in reporting. For further information on Nature Portfolio policies, see our Editorial Policies and the Editorial Policy Checklist.

Statistics

For all statistical analyses, confirm that the following items are present in the figure legend, table legend, main text, or Methods section.

n/a	Confirmed	
☐	☒	The exact sample size (*n*) for each experimental group/condition, given as a discrete number and unit of measurement
☐	☒	A statement on whether measurements were taken from distinct samples or whether the same sample was measured repeatedly
☐	☒	The statistical test(s) used AND whether they are one- or two-sided<br>*Only common tests should be described solely by name; describe more complex techniques in the Methods section.*
☒	☐	A description of all covariates tested
☐	☒	A description of any assumptions or corrections, such as tests of normality and adjustment for multiple comparisons
☐	☒	A full description of the statistical parameters including central tendency (e.g. means) or other basic estimates (e.g. regression coefficient) AND variation (e.g. standard deviation) or associated estimates of uncertainty (e.g. confidence intervals)
☐	☒	For null hypothesis testing, the test statistic (e.g. *F*, *t*, *r*) with confidence intervals, effect sizes, degrees of freedom and *P* value noted<br>*Give P values as exact values whenever suitable.*
☒	☐	For Bayesian analysis, information on the choice of priors and Markov chain Monte Carlo settings
☒	☐	For hierarchical and complex designs, identification of the appropriate level for tests and full reporting of outcomes
☐	☒	Estimates of effect sizes (e.g. Cohen's *d*, Pearson's *r*), indicating how they were calculated

Our web collection on statistics for biologists contains articles on many of the points above.

Software and code

Policy information about availability of computer code

Data collection	Behavioural data were recorded using Unity (ver 2020.3.14f1), and SteamVR plugin (2.7.3; SDK 1.14.15), eye movements with SRanipal (v1.1.0.1)
Data analysis	MATLAB (v2022a), JASP (0.16.3.0), and the psignifit toolbox (https://github.com/wichmann-lab/psignifit/wiki). Custom analysis code is available<br>for download at https://osf.io/8djtq/.

For manuscripts utilizing custom algorithms or software that are central to the research but not yet described in published literature, software must be made available to editors and reviewers. We strongly encourage code deposition in a community repository (e.g. GitHub). See the Nature Portfolio guidelines for submitting code & software for further information.

Data

Policy information about availability of data

All manuscripts must include a data availability statement. This statement should provide the following information, where applicable:

- Accession codes, unique identifiers, or web links for publicly available datasets
- A description of any restrictions on data availability
- For clinical datasets or third party data, please ensure that the statement adheres to our policy

The behavioural data generated in this study have been deposited in the public database under accession code https://osf.io/8djtq/.

Research involving human participants, their data, or biological material

Policy information about studies with human participants or human data. See also policy information about sex, gender (identity/presentation), and sexual orientation and race, ethnicity and racism.

Reporting on sex and gender	Thirty-six human adults (22 females, 14 males) participated in the experiment
Reporting on race, ethnicity, or other socially relevant groupings	No such groupings were used.
Population characteristics	Thirty-six human adults (Mean age = 19.6, SD = 2.6), with normal or corrected to normal vision.
Recruitment	Participants were recruited via convenience sampling from the University of Sydney undergraduate psychology cohort and received either course credit or 20 AUD per hour of their time. They were informed of the option to opt-out at any time without penalty, and we can foresee no self-selection biases that could have meaningfully influenced our results.
Ethics oversight	The experiment was approved by The University of Sydney Human Research Ethics Committee.

Note that full information on the approval of the study protocol must also be provided in the manuscript.

Field-specific reporting

Please select the one below that is the best fit for your research. If you are not sure, read the appropriate sections before making your selection.

☒ Life sciences ☐ Behavioural & social sciences ☐ Ecological, evolutionary & environmental sciences

For a reference copy of the document with all sections, see nature.com/documents/nr-reporting-summary-flat.pdf

Life sciences study design

All studies must disclose on these points even when the disclosure is negative.

Sample size	The sample size was chosen to exceed the basis of prior studies that have examined perceptual oscillations in behaviour (e.g. n=16; Landau & Fries, 2012).
Data exclusions	We recruited 45 participants in total, 7 of whom were excluded for incomplete data collection that either resulted from wireless signal dropout or hardware malfunction. One more was excluded on the basis of eye-movement data, and another for an error in eye-movement calibration.
Replication	The experiment was performed independently once. We calculated the within-participant replication probability of all group-level effects, using Bayesian estimates of population prevalence (Inceet al.,2021). All effects had high replication probability within the sample.
Randomization	All experimental parameters, such as block order, stimulus timing etc, were randomized within and across participants.
Blinding	No blinding was used during this experiment, because a within-participant analysis is the main focus.

Reporting for specific materials, systems and methods

We require information from authors about some types of materials, experimental systems and methods used in many studies. Here, indicate whether each material, system or method listed is relevant to your study. If you are not sure if a list item applies to your research, read the appropriate section before selecting a response.

Materials & experimental systems

n/a	Involved in the study
☒	☐ Antibodies
☒	☐ Eukaryotic cell lines
☒	☐ Palaeontology and archaeology
☒	☐ Animals and other organisms
☒	☐ Clinical data
☒	☐ Dual use research of concern
☒	☐ Plants

Methods

n/a	Involved in the study
☒	☐ ChIP-seq
☒	☐ Flow cytometry
☒	☐ MRI-based neuroimaging

Plants

Seed stocks	n/a
Novel plant genotypes	n/a
Authentication	n/a
